# A Review of Artificial Intelligence-Based Dyslexia Detection Techniques

**DOI:** 10.3390/diagnostics14212362

**Published:** 2024-10-23

**Authors:** Yazeed Alkhurayyif, Abdul Rahaman Wahab Sait

**Affiliations:** 1 Department of Computer Science, College of Computer Science, Shaqra University, Shaqra 11961, Saudi Arabia; 2 Department of Documents and Archive, Center of Documents and Administrative Communication, King Faisal University, Al-Ahsa 31982, Saudi Arabia

**Keywords:** feature extraction, feature selection, dimensionality reduction, deep learning, machine learning, dyslexia identification, convolutional neural network

## Abstract

Problem: Dyslexia is a learning disorder affecting an individual’s ability to recognize words and understand concepts. It remains underdiagnosed due to its complexity and heterogeneity. The use of traditional assessment techniques, including subjective evaluation and standardized tests, increases the likelihood of delayed or incorrect diagnosis. Motivation: Timely identification is essential to provide personalized treatment and improve the individual’s quality of life. The development of artificial intelligence techniques offers a platform to identify dyslexia using behavior and neuroimaging data. However, the limited datasets and black-box nature of ML models reduce the generalizability and interpretability of dyslexia detection (DD) models. The dimensionality reduction technique (DRT) plays a significant role in providing dyslexia features to enhance the performance of machine learning (ML)- and deep learning (DL)-based DD techniques. Aim: In this review, the authors intend to investigate the role of DRTs in enhancing the performance of ML- and DL-based DD models. Methodology: The authors conducted a comprehensive search across multiple digital libraries, including Scopus, Web of Science, PubMed, and IEEEXplore, to identify articles associated with DRTs in identifying dyslexia. They extracted 479 articles using these digital libraries. After an extensive screening procedure, a total of 39 articles were included in this review. Results: The review findings revealed various DRTs for identifying critical dyslexia patterns from multiple modalities. A significant number of studies employed principal component analysis (PCA) for feature extraction and selection. The authors presented the essential features associated with DD. In addition, they outlined the challenges and limitations of existing DRTs. Impact: The authors emphasized the need for the development of novel DRTs and their seamless integration with advanced DL techniques for robust and interpretable DD models.

## 1. Introduction

Dyslexia is a neurodevelopmental disorder causing challenges to individuals in terms of word recognition, spelling, and decoding [1]. It is one of the common learning abilities, affecting 5–10% of the global population [2,3]. The academic environment can be challenging for dyslexic individuals (DIs). Identifying dyslexia in the earlier stages can protect individuals from adverse effects, including reduced quality of life, low self-esteem, and frustration [4]. Diagnosing dyslexia demands clinical, educational, and behavioral assessments [5]. Traditionally, trained professionals conduct standardized tests of reading fluency, phonological awareness, and rapid automatized naming. Integrating brain imaging, cognitive assessments, language studies, and behavioral observations is essential for identifying dyslexia [5]. Although resource-intensive, time-consuming, and ambiguous, these assessments provide valuable insights into dyslexia identification.

Potential sources of dyslexia detection (DD) include standardized psycho-educational assessments, web/mobile games, eye movement monitoring, neuroimaging, and video/images [6]. In addition, functional magnetic resonance imaging (MRI) and diffusion tensor imaging reveal dyslexic and non-dyslexic brain structure and function [7]. Functional MRI may indicate brain activity during reading tasks, whereas structural MRI can show brain anatomical changes, including gray matter volume or cortical thickness [8]. Dyslexia-related brain activity and connectivity may be captured using electroencephalogram (EEG) signals [9]. Reading, phonological awareness, and working memory assessments investigate cognitive and linguistic skills related to reading and language processing [9]. Genome-wide association studies and candidate gene methods are utilized to uncover dyslexia-risk genes.

Recently, computational methods have been widely used to improve DD. Integrating dimensionality reduction techniques (DRTs) with machine learning (ML) approaches presents an opportunity for building objective and automated screening tools [10]. These methods identify discriminative patterns and biomarkers in neuroimaging, behavioral, and linguistic data. Machine learning (ML) and deep learning (DL) models employ DRTs to minimize the number of features in a dataset while preserving crucial information [11]. A broader spectrum of dyslexia patterns can be captured using high-dimensional data. Model overfitting is the primary challenge associated with these data types [11]. This may limit the model’s generalizability, leading to false positive outcomes. A huge computational cost is required to process high-dimensional data [11]. In addition, the lack of feature interpretation hinders the model’s ability to gain insights into dyslexia’s mechanisms. DRTs address the curse of dimensionality [11]. It simplifies data representation, reduces features, improves model interpretability, and increases computing performance. Reduced dimensionality assists in recognizing key features and comprehending dyslexia detection algorithms [12].

Feature extraction and selection are the primary approaches of DRTs [13]. The objective of feature selection is to identify a subset of primary features of dyslexia. Feature extraction transforms the primary features into a novel set of features, capturing the essential information of dyslexia [13]. These techniques can extract relevant patterns and biomarkers from neuroimaging images, behavioral evaluations, and language analyses [14].

DRTs include principal component analysis (PCA), t-distributed stochastic neighbor embedding (t-SNE), independent component analysis (ICA), singular value decomposition (SVD), uniform manifold approximation, and linear discriminant analysis (LDA), which are widely applied for identifying crucial features. PCA distributes data into a lower-dimensional subspace by identifying its principal components. It captures global trends and reduces noise in high-dimensional data [12,13]. The t-SNE technique effectively captures local structures and reveals data clusters. It is used in exploratory data analysis. Independent component analysis divides the multivariate signals into additive and independent components. It is used to identify brain activity patterns. SVD factorizes a matrix into multiple matrices, revealing the underlying structure of the data. It is commonly applied in developing natural language processing-based applications. Uniform manifold approximation is a non-linear DRT, preserving local and global structures within the data. It visualizes the relationships among complex data. LDA is a supervised DRT detecting a linear combination of features, separating multiple classes in the data. With advances in ML, neuroimaging, and computational linguistics, dyslexia diagnoses are increasingly common. Convolutional neural networks (CNNs) are widely used for extracting features from medical images. These models can reduce the feature dimensions using their inherent features.

Researchers can better understand dyslexia and develop multidisciplinary strategies to improve diagnosis and intervention by integrating results from multiple domains. The findings of a systematic review on dyslexia detection can encourage cross-disciplinary collaboration and information sharing among academics, clinicians, educators, and policymakers. The findings of existing reviews [7,8,9,10,11,12,13,14,15] highlighted the significance of extracting biomarkers in detecting dyslexia. These studies lacked a detailed analysis of DRTs in enhancing ML- and DL-based models. There is a gap concerning the role of DRTs in detecting dyslexia using diverse data. Recent advancements in artificial intelligence (AI) underscore the importance of DRTs in improving the DD model’s performance and interpretability. Researchers frequently develop and enhance DRTs for DD. Innovative DRTs can reveal dyslexia biomarkers and patterns, improving detection algorithms’ accuracy, speed, and dependability. These factors motivated the authors to conduct a systematic literature review of dyslexia detection techniques. Thus, this study intends to synthesize DRTs related to dyslexia detection. It focuses on diverse classification techniques for identifying dyslexia. It provides the potential of DRTs in addressing the unique challenges associated with high-dimensional data in dyslexia research. In addition, it presents the existing challenges and limitations of data collection, dimensionality reduction, and classification techniques.

The study is structured as follows: Section 2 highlights the methodology of the review. The outcomes of the review are outlined in Section 3. Section 4 discusses the significance of the review findings. Lastly, Section 5 presents the review’s contributions and limitations.

## 2. Review Methodology

The Preferred Reporting Items for Systematic Reviews and Meta-Analyses (PRISMA) guidelines [16] were followed to present a structured framework highlighting the significance of FES technologies in detecting dyslexia using multiple modalities. Based on the research objectives, the authors developed a set of research questions.

**Research Question 1 (RQ1):** How do DRTs improve dyslexia detection?

This question aims to find the role of DRTs in differentiating dyslexic and non-dyslexic individuals. It identifies the significance of the multiple modalities on dyslexia models’ performance and generalizability.

**Research Question 2 (RQ2):** What are the crucial biomarkers associated with dyslexia?

RQ2 explores the identification of biomarkers associated with dyslexia across multiple modalities. It uncovers the neurobiological and linguistic characteristics associated with dyslexia.

**Research Question 3 (RQ3):** What are the challenges and opportunities in extracting dyslexia features and developing DD models?

RQ3 presents the methodological challenges encountered in dyslexia detection using DRTs. It evaluates the impact of these challenges and limitations on model generalizability.

### Search Strategies

The authors employed a comprehensive list of keywords relevant to dyslexia detection, feature extraction, and selection. The terms included dyslexia, feature extraction, feature selection, machine learning, deep learning, behavioral assessments, linguistic analyses, and neuroimaging. In addition, the authors considered synonyms, variations, and related terms in order to ensure detailed coverage of the literature.

Academic databases associated with dyslexia research, computational neuroscience, psychology, computer science, and linguistics were used in this study. The authors considered PubMed, Web of Science, Scopus, IEEEXplore, and ACM digital library databases to extract the peer-reviewed literature. They constructed search strings using Boolean operators (AND, OR, and NOT) to combine keywords logically. The syntax of the strings was customized according to the databases in order to maximize the retrieval of relevant articles. Table 1 outlines the inclusion and exclusion criteria with search strings.

**Figure 1:**
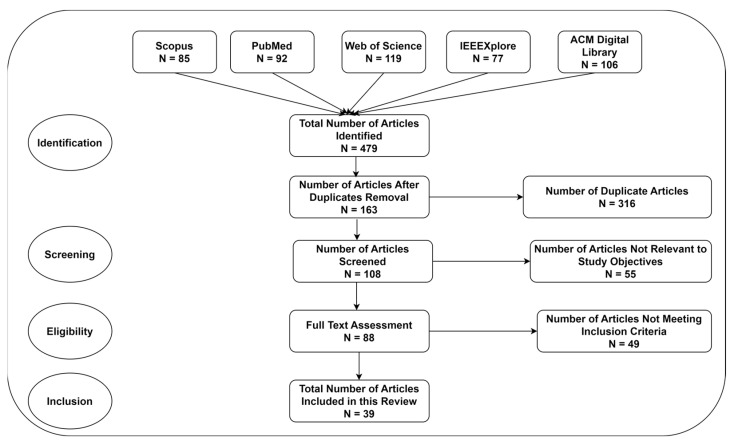
Extraction of research articles.

Figure 1 highlights the extraction of research articles using the PRISMA guidelines. Based on the assessment of the title and abstract, the authors extracted a set of articles for a full assessment. Table 2 presents the quality assessment techniques to guarantee the reliability and validity of the studies. The authors evaluated each article to confirm its alignment with the objectives of this review. Each article was evaluated using these criteria, yielding a total score of 15 (3 points for each criterion). The authors extracted data from the included studies, focusing on key aspects, including research methodology, participant demographics, authors, publication year, type of DRT and classifier, and limitation. Multiple DRTs and modalities and their impact on the model’s generalizability were explored. Throughout the review process, they minimized bias using independent assessments. They resolved the discrepancies through discussion and consensus. In order to improve the selection process, the authors recruited three healthcare experts to evaluate the significance of the studies in clinical settings. The results were synthesized narratively.

## 3. Results

Figure 1 shows the findings of the literature extraction. A total of 479 studies were extracted using the suggested search strategies. After the removal of duplicates and screening abstracts, 88 articles were identified for full-text assessment. Subsequently, a rigorous assessment was conducted using the inclusion and exclusion criteria. Finally, the authors included 39 articles in this review.

**Figure 2:**
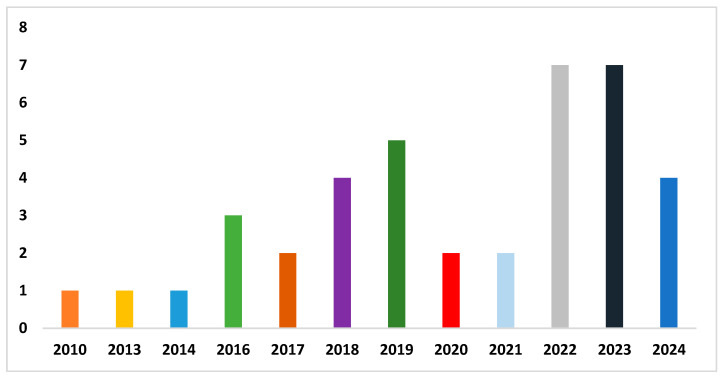
Number of DD studies published per year.

**Figure 3:**
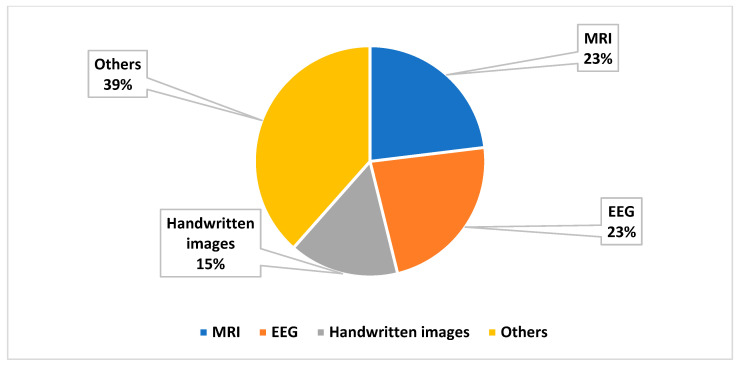
Classification of DD based on modalities.

Figure 2 presents the publication years of the included studies. A larger number of studies were published between 2019 and 2024. This indicates the significance of artificial intelligence in detecting dyslexia. Figure 3 presents the studies based on multiple modalities. A significant number of studies were based on MRI and EEG. In addition, various data sources, including eye movement, gamified data, etc., were used for DD.

**Figure 4:**
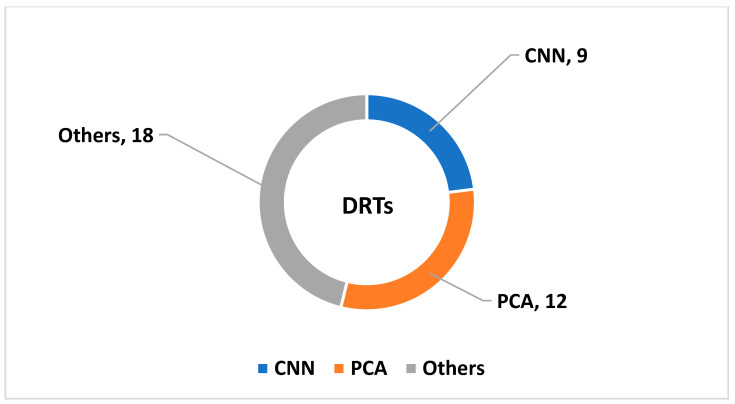
Classification of DD based on the DRTs.

Figure 4 presents the studies based on the DRTs. PCA and convolutional neural network (CNN) techniques were widely applied for feature extraction and selection. Particle swarm optimization (PSO) and random forest (RF) were widely employed to identify key features.

**Figure 5:**
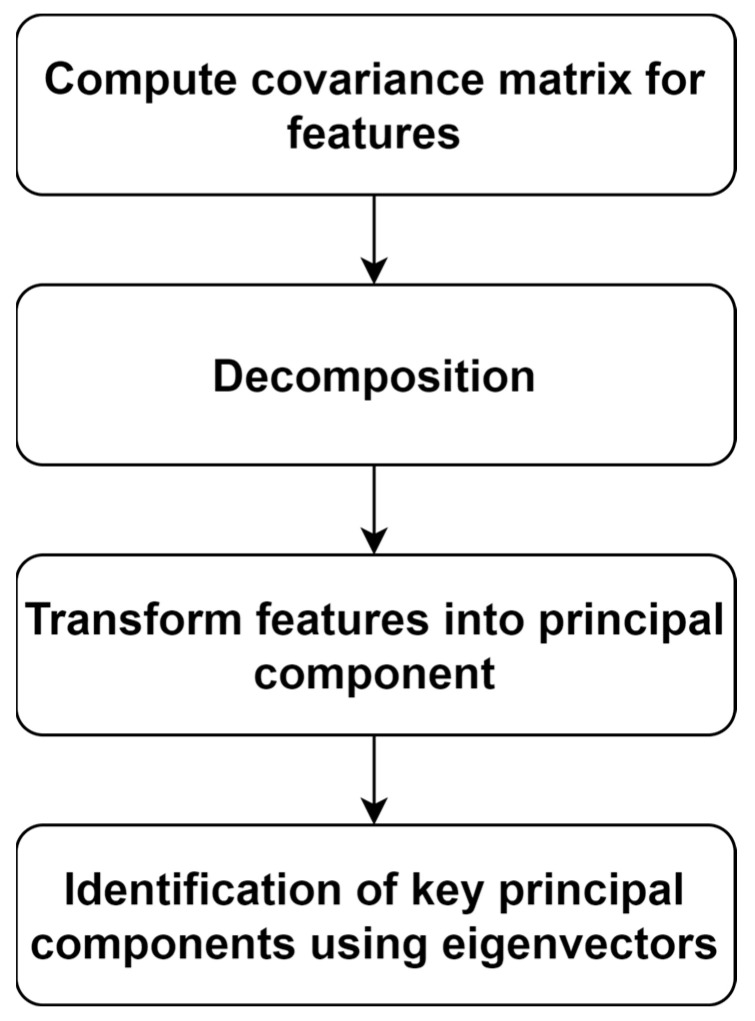
Feature extraction using PCA.

PCA computes the covariance matrix for features. Eigenvectors play a crucial role in extracting key features. However, the non-linearity among the features may reduce the efficiency of PCA. Additionally, the interpretation of extracted features may not be straightforward. The process of extracting features using PCA is presented in Figure 5.

**Figure 6:**
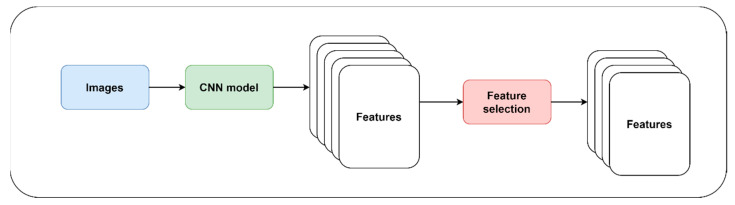
Feature extraction and selection using CNN.

Figure 6 shows the CNN-based feature extraction and selection. CNN models are not an explicit DRT. However, they inherently reduce the dimensions of the features using convolution and pooling layers. CNN models were primarily used to extract features from MRI, handwritten images, and EEG signals. Feature importance techniques were used in building DD that were capable of interpreting the features.

The included studies covered diverse populations, including children and adults with dyslexia. The FMRI studies highlighted the significance of brain activation patterns in detecting abnormal individuals. Similarly, behavioral assessments were crucial in detecting dyslexia in the initial stages. For instance, a significant difference was observed in individuals with dyslexia during the experimental tasks, including phonological processing skills, rapid naming, and reading fluency. The authors addressed RQ1, RQ2, and RQ3 by identifying the strengths and weaknesses of the included studies. The following section presents the in-depth solutions to the research questions.

### 3.1. DRT-Based Dyslexia Detection

The authors classified the studies based on the DRT used for dyslexia detection. Figure 3 presents the DRT used for dyslexia detection. Initially, data were pre-processed in order to filter the outliers or irrelevant patterns. Feature extraction techniques or tools, including the FreeSurfer version 7.1.1 application, Online Games, PCA, and CNN, were employed to extract crucial features. In addition, feature selection techniques such as leave-one-out cross-validation (LOOCV), PSO, and search optimization techniques were used to identify significant features of dyslexia.

#### 3.1.1. PCA-Based DD Models

Table 3 reveals the features and limitations of PCA-based DD models. It presents the datatypes and performance of each study. Al-Barhantesly and Motawah [17] employed the EEG dataset to identify dyslexia. They employed Fourier transform analysis and statistical functions to extract features. An artifact subspace reconstruction (ASR)-based PCA was used to identify critical features associated with dyslexia. An SVM classifier was used to classify the extracted features into normal and abnormal individuals. Asvestopoulou et al. [18] integrated feature extraction and selection techniques to identify unique features of dyslexia using eye movements. They analyzed the eye-tracking data of 135 individuals and classified them using the SVM classifier. Pre-trained CNN models were used to extract features. The region of interest was used to select the crucial features. Appadurai and Bhargavi [19] used the eye-tracking dataset for dyslexia detection. They applied PCA for feature extraction. Particle swarm optimization (PSO) was used to assign weights for each feature. The SVM classifier with a PSO-optimized kernel was used to detect dyslexia. Raatikainen et al. [20] used eye movement data for DD. They applied a transition matrix with a histogram for the feature extraction. They extracted 246 features and selected the unique features using the random forest (RF) algorithm.

Christodoulides et al. [21] used the PCA for feature extraction. The EEG signal features were classified using the RF technique. The model achieved a classification accuracy greater than 95%. Parmar et al. [22] utilized EEG signals to detect dyslexia. They applied PCA to extract critical features. However, the limitations of PCA reduced the overall performance of the SVM, leading to a low accuracy of 79.3%. Liyakathunisa et al. [23] used PCA and XGBoost techniques to identify unique features of dyslexia. They developed an NN model to classify the features into dyslexia and non-dyslexia. Parmar and Paunwala [24] developed a DD model using SVM. They use t-SNE for pre-processing the data. PCA was used to reduce the feature dimension. Zaree et al. [25] built an ensemble learning model to identify dyslexia using event-related potential (ERP). They used ICA and PCA for processing and selecting key dyslexia patterns. Zhong et al. [26] introduced a multi-level feature extraction technique to extract multiple strokes of Chinese characters. They employed the XGBoost model to classify the features. A tablet application was developed to assess children’s cognitive ability. The model achieved an accuracy of 81.14% with an area under the receiver operating characteristic curve (AUC) of 0.79. El-Hmimdi et al. [27] used eye movement data to detect dyslexia. They used SVD and PCA for selecting the features. They classified the features using a CNN model. Shalileh et al. [28] employed multi-layer perceptron to classify eye movement data. LDA was used to identify the unique dyslexia features. The feature dimensions were reduced using PCA.

#### 3.1.2. CNN-Based DD Models

The performance of the CNN-based DD models is presented in Table 4. Usman and Muniyandi [29] employed a T1-weighted MRI of 45 individuals to extract biomarkers of dyslexia. They constructed deep CNN with a weightage assignment system for extracting features from encrypted MRI. A CNN model was developed to classify the extracted features. Tomaz Da Silva et al. [30] constructed a CNN model for feature extraction. They applied guided back-propagation to identify crucial features. A modified LeNet-5 with ReLU activation was used to classify dyslexia. Sangeetha et al. [31] extracted MRI features using a CNN model. They employed an ML model to classify the extracted features. Harismithaa and Sudha Sadasivam [32] proposed a DD model using CNN and time-distributed convolutional long-short-term memory (LSTM). They employed a CNN model to extract features from the neuro-imaging data. Multi-modal features were integrated using a fusion technique. Sasidhar et al. [33] employed a CNN model pre-trained to key features of handwritten images in order to differentiate dyslexic and non-dyslexic individuals.

Kothapalli et al. [34] introduced a multi-modality-based DD. They employed recurrent neural network (RNN) and CNN models for feature extraction. An ensemble learning approach was followed to classify the extracted features. The decision tree was used as a meta-learner to make decisions using the outcomes of RF and NB models. Ileri et al. [35] constructed a one-dimensional CNN model to classify EOG signals into healthy and unhealthy classes. They applied a segmentation technique to divide the signals into multiple features. A pair of convolutional layers with classification function were used for signal classification. Jasira and Laila [36] used the CNN model to extract features. An LSTM model was used to classify the features. Liu et al. [37] developed a hybrid feature extraction technique with model interpretation for detecting dyslexia.

#### 3.1.3. Other DRT Models

Table 5 reveals the characteristics of studies related to other DRT models. Most studies did not fully leverage the potential of DRTs in order to capture the crucial patterns associated with DD. Deans et al. [38] conducted a study to identify reading disabilities. They used a viewpoint eye tracker application to extract features of an individual’s eye movements. Linear regression was used for the feature classification. Fred and Breznitz [39] used the ERP signals of 32 individuals. They used discrete Fourier transform and fast Fourier transform to extract statistical and spatial features. In order to identify unique features, they used a spectral flatness measure, spectral roll-off, spectral centroid, and power spectral density. Karim et al. [40] applied a kernel density estimation in order to extract meaningful dyslexia features. Plonski et al. [41] used the FreeSurfer image analysis approach to extract crucial features of dyslexia. The techniques, including descending importance and log-loss value, were used to select the critical features. However, the classifier obtained a lower accuracy of 65% with an AUC of 66%. Cui et al. [42] extracted specific white matter regions using a multi-level ML approach. They applied linear SVM for feature classification and achieved an accuracy of 83.61%. Benfatto et al. [43] used the dynamic dispersion threshold algorithm to extract crucial features. Maximum margin with linear SVM was used to select unique features and classify dyslexic and non-dyslexic samples. The authors applied the recursive feature elimination technique to improve the classifier accuracy. Tamboer et al. [44] obtained a classification accuracy of 80% using Jacobian vector-based feature extraction. Plonski et al. [45] used the FreeSurfer application for feature extraction. They used multiple classifiers to identify dyslexia.

Khan et al. [46] used the performance score of an online assessment for DD. They developed an ML model to classify normal and dyslexic individuals. The performance of the ML model was better compared to that of human experts. Rello et al. [47] built an interactive game application to evaluate the performance of individuals. They designed the application to collect data, including linguistic and cognitive skills. The features were extracted using the gaming interface. The SVM classifier was used to classify the features. Perera et al. [48] used the EEG dataset to find unique dyslexia patterns. They extracted crucial EEG channels associated with dyslexia using the ASR technique. They employed sub-band decomposition to analyze the EEG signals. The SVM classifier was used for binary classification. Rezvani et al. [49] used the EEG dataset to train a model to detect dyslexia. Using the segmentation technique, a brain vision analyzer was used to remove artifacts and extract features. Weighted connectivity measures were applied to select the significant features related to reading abilities.

Spoon et al. [50] employed the handwritten images of individuals to detect dyslexia. The Tesseract model was used to extract features. The extracted features were classified using a CNN model. The authors conducted 10-fold cross-validation to evaluate the model. The model achieved an average accuracy of 55.7%. In another study, Spoon et al. [51] fine-tuned a CNN model using hyperparameter optimization and achieved an accuracy of 77.6% using handwritten images. Zahia et al. [52] used FMRI images to classify dyslexic and non-dyslexic individuals. They employed statistical parametric maps for the feature extraction. A 3D CNN model was used to classify the extracted features. They evaluated the model’s generalizability using four-fold cross-validation. The model obtained an average accuracy of 72.73% with an F1-score of 67%. Zainuddin et al. [53] employed discrete wavelet transformation for EEG feature extraction. An extreme machine learning model was used to classify the features. Seshadri et al. [54] employed digital wavelet transform (DWT) to extract EEG features. A filter-based feature selection method was used to identify unique features. A shallow deep NN model was constructed to detect dyslexia. Gasmi et al. [55] used a gamified dataset to detect dyslexia. They employed a filter function to extract features from the gamified data. An ensemble learning approach was used to classify the features.

**Table 5:** Other DRT models.

Authors	Data Type	Type of DRT	Classifier	Dataset Size (Number of Individuals)	Performance	Limitations
Deans et al. (2010) [39]	Eye movements	Viewpoint eye tracker	LR	77	Accuracy: 78.2%	Eye movement tasks caused excessive eye movements in participants. These tasks may affect the research findings.
Frid and Breznitz (2012) [42]	ERP	Discrete Fourier transformation and ML model	ML model	32	Accuracy: 86.3%	Complex data, including ERPs, require human intervention to analyze nuanced patterns, and automated analysis can overlook essential dyslexia patterns.
Karim et al. (2013) [25]	EEG	Kernel density estimation	MLP	52	Eye-close accuracy: 98.2%, eye-open accuracy: 94.3%	The limitations of MLP may influence the model’s generalization.
Plonski et al. (2014) [16]	MRI	Freesurfer image analysis, descending importance technique, and LOOCV	LR	236	Accuracy: 65%, AUC: 0.66	Data dependency on the site location and limited data acquisition processes reduced the effectiveness of the model.
Cui et al. (2016) [17]	MRI	Leave-one-out cross-validation	Linear SVM	61	Accuracy: 83.21%	A limited sample size may reduce the model’s generalizability.
Benfatto et al. (2016) [40]	Eye movements	Dynamic dispersion threshold	Maximum-margin SVM	2165	Accuracy: 95.6%, specificity: 95.5%, sensitivity: 95.77%	Limited information related to the impact of language on eye movement patterns and reading difficulties.
Tamboer et al. (2016) [19]	MRI	Jacobian vector approach	Linear SVM	109	Accuracy: 80%	Focusing on specific brain regions may ignore other dyslexia-related brain regions.
Plonski et al. (2017) [18]	MRI	Freesurfer image analysis and LOOCV	SVM, RF, and LR	236	AUC: 0.66	Data splitting based on gender decreased the reliability of the findings.
Khan et al. (2018) [41]	Behavioral data	Online test-based feature extraction	ML model	857	Accuracy: 99%	Feature selection transparency is essential to comprehending the model’s decision-making process and capturing dyslexia characteristics.
Rello et al. (2018) [43]	Behavioral data	Game-based feature extraction	SVM	267	Accuracy: 84.62%	Clinical interpretation requires an understanding of dyslexia’s cognitive processes.
Perara et al. (2018) [27]	EEG	ASR	SVM	80	Accuracy: 95%, sensitivity: 88.24%, specificity: 66.67%	Lack of interpretability of EEG features associated with dyslexia.
Rezvani et al. (2019) [28]	EEG	Brain vision analyzer	SVM and KNN	44	Accuracy: 95%	Group imbalance may influence the study outcomes.
Spoon et al. (2019) [33]	Handwritten images	Tesseract-based feature extraction	CNN	100	Accuracy: 55.7%	Lack of diversity in samples may hinder generalizability.
Spoon et al. (2019) [34]	Handwritten images	Tesseract-based feature extraction	CNN	100	Accuracy: 77.6%	
Zahia et al. (2020) [20]	MRI	Statistical parametric maps	3D CNN	55	Accuracy: 72.73%, F1-score: 67%	Reliable data quality requires conversion to Nifti volumes, head motion compensation, normalization, and smoothing. These stages may cause unpredictability and biases with a lack of preprocessing techniques.
Zainuddin et al. (2022) [29]	EEG	DWT	Extreme learning machine	36	Accuracy: 88%	Lack of effective pre-process, leading to limited performance.
Seshadri et al. (2023) [32]	EEG	DWT	NN	20	Accuracy: 97.5%	A limited number of samples may reduce the model’s generalizability.
Gasmi et al. (2024) [50]	Behavioral data	Web-based game	Ensemble learning-based model	3644	Accuracy: 90.15%	The model’s performance was limited to specific web games. The generalization of the model for a diverse population is different.

### 3.2. Dyslexia Biomarkers

**Figure 7:**
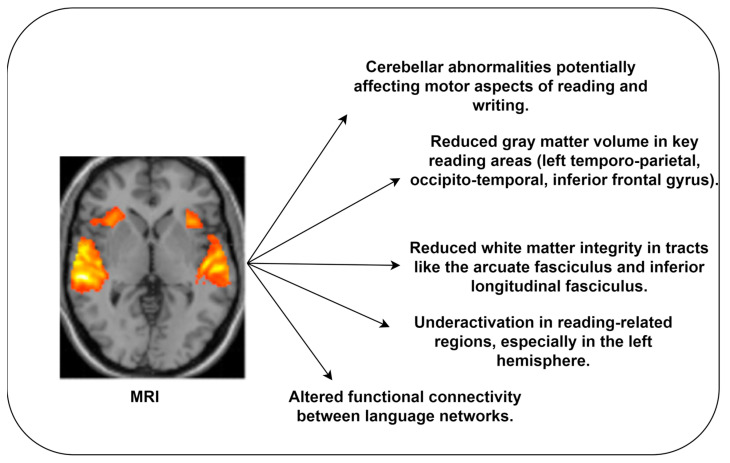
Dyslexia biomarkers extracted from MRI.

The neurological underpinnings of dyslexia were revealed through the findings of the included studies. Figure 7 highlights the key dyslexia biomarkers extracted from MRI.

Functional MRI offer valuable insights into the functionality of the brain of an individual during a specific task. Dyslexic individuals frequently exhibit reduced functionality in the brain regions. By extracting biomarkers, clinicians can identify dyslexia in earlier stages. MRI-based DD studies [6,30,32] highlighted the structural differences in the brains of dyslexic individuals compared to normal individuals. These studies reported the significance of reduced gray matter volume in the left temporoparietal cortex, left occipitotemporal cortex, and left inferior frontal gyrus in dyslexic individuals.

Variations in white matter tracts, including arcuate fasciculus and superior longitudinal fasciculus, are associated with cognitive impairment. These variations may influence the individual’s information processing speed, neural connectivity, and communication between brain regions related to reading and language comprehension. Resting-state FMRI studies reported functional connectivity, including disruptions in interaction between language and sensory-motor regions in dyslexic individuals. The altered functional connectivity may affect the ability to integrate information for reading and language processing. In addition, the studies highlighted atypical asymmetry patterns in brain regions associated with phonological processing and auditory perception.

Extracting features from handwritten images in order to identify deviations from standard letter forms is a widespread practice differentiating dyslexic and non-dyslexic individuals. Inconsistencies in size, slope, orientation, closure (open versus closed loops), and excessive strokes are the key features associated with dyslexia. The gap between letters and words or irregular baselines may indicate difficulties in motor planning and spatial organizing. Dyslexics may write crowdedly, blurred, or with inadequate margins. Smoothness and flow may be assessed in handwriting samples. Slow motions, jerky movements, and frequent erasures may indicate motor planning and processing disorders.

**Figure 8:**
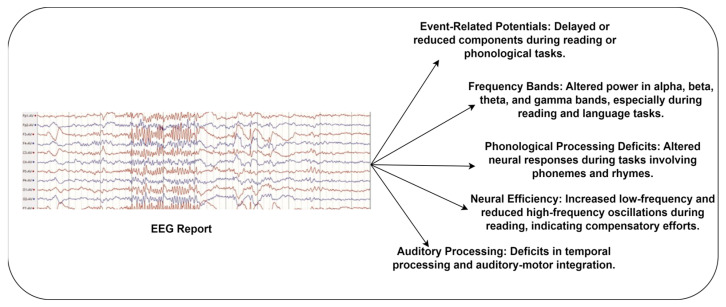
Dyslexia biomarkers extracted from EEG.

Diagnosing dyslexia using EEG data involves studying time-domain, frequency-domain, connectivity, non-linear, statistical, and graph theory aspects. Each component provides unique insights into dyslexia’s brain dynamics and cognitive processes, offering a rigorous foundation for accurate identification and a better understanding of this complex learning disorder. The included studies [21,22,48,49,54] analyzed EEG signals to detect dyslexia by capturing brain dynamics and cognitive processes. EEG signal amplitude and latency are time-domain features. Variations in these features may suggest dyslexia-related brain activity. The authors of [42] extracted multiple ERP features associated with dyslexia. Dyslexics exhibit variable amplitude and latency of the P300 component, which is connected with attention and working memory. Similarly, the N400 component presents the existence of challenges related to language processing and semantic comprehension. In addition, mismatch negativity indicates dyslexia-related auditory processing and phonological awareness impairments. Figure 8 reveals the critical biomarkers for identifying dyslexia using EEG.

Saccades, regressions, and fixation length are significant eye movements. Fixation periods are greater in DIs, suggesting visual processing difficulties. DIs have longer and more frequent saccades, which causes challenges in switching between words or lines of text. Increased regressions, or eye movements to previously read text, indicate difficulties in decoding and processing textual content. Additionally, word fixations and reading duration may quantify DI reading habits and efficiency.

### 3.3. Challenges and Opportunities

DRT minimizes the number of features without losing relevant information [37]. However, dimensionality reduction may not completely capture the complexity of the original data, resulting in a degree of information loss. Loss of information may contribute to the absence of crucial dyslexia characteristics or patterns, compromising detection models. DRT may increase model interpretability by lowering the number of features. Nevertheless, it may cause challenges in interpreting the reduced features. Understanding dyslexia’s cognitive and neurological underpinnings are essential for designing effective detection models and therapies. Researchers may struggle to assess the biological and cognitive implications of the extracted features.

Privacy, informed consent, and algorithmic bias should be considered in dyslexia detection studies. Scientific integrity requires openness, impartiality, and accountability in data collection, analysis, and interpretation. Researchers have to comply with ethical norms to protect dyslexia research subjects. Assessing dyslexia detection algorithms’ applicability to educational and clinical contexts is challenging. Successful implementation requires adapting models to varied people, environmental circumstances, and practical constraints. Furthermore, robust research designs and cooperation with educational and clinical stakeholders are essential to validate DD models in real-world settings.

Researchers can use feature visualization and mapping systems to analyze extracted features and understand dyslexia-related processes. These methods may boost the confidence and transparency of the DD model by strengthening the model’s interpretability. DRT may be improved to combine and evaluate neuroimaging, behavioral, linguistic, and genetic marker data. Multi-view learning and joint embedding approaches may help identify dyslexia-related patterns by combining diversified data sources. Dynamic component analysis and dynamic mode decomposition may reveal spatiotemporal differences in dyslexia-related data and reveal changing patterns throughout developmental stages. Dynamic DRT can enhance dyslexia progression characterization and prediction by accounting for temporal dynamics. Multimodal fusion techniques in dyslexia detection studies are essential for progress and clinical application.

Personalized intervention, adaptive learning, and assistive technology are possible suggestions using machine learning models. Targeting dyslexia identification and treatments to individual needs and learning profiles improves patient outcomes. Personalized intervention strategies in dyslexia research may improve an individual’s quality of life. In dyslexia research, data sharing, code repositories, and collaborative platforms improve transparency, reproducibility, and knowledge exchange. Benchmarking challenges and open datasets boost innovation and advancements in the field. Open science in dyslexia detection research promotes cooperation, discovery, clinical practice, and policy impact.

Sparse and structured DRTs can enforce restrictions on learned representations to promote sparsity, interpretability, and domain-specific structure. Sparse PCA, group LASSO, and graph-based regularization can identify sparse and organized dyslexia features from multi-modal data. These strategies improve dyslexia biomarker interpretability and diagnostic relevance by improving sparsity and structure. Modeling using domain-specific information and prior knowledge improves dimensionality reduction. Understanding dyslexia-related brain areas, cognitive processes, and behavioral characteristics may aid DRTs. Bayesian methods, knowledge-based restrictions, and domain-specific regularization terms may incorporate domain knowledge into dimensionality reduction models.

Interdisciplinary collaboration is essential to understand the dyslexia condition and develop effective interventions. Several factors, including cognitive, linguistic, genetic, and environmental, are associated with dyslexia. Thus, an integrated approach using these disciplines can provide a holistic solution to treat dyslexia. For instance, neuroscientists can explore dyslexia’s brain mechanisms using MRI and EEG data. Similarly, psychologists and behavioral scientists can analyze dyslexia’s emotional and psychological impacts and provide therapies to improve academic performance and mental health. By leveraging these disciplines, dyslexia research can yield effective approaches to detect dyslexia in its initial stages.

## 4. Discussions

By leveraging the PRISMA guidelines, the authors presented in-depth information on DRT-based dyslexia detection. The authors addressed the research questions by extracting the essential strategies related to DD. According to the review findings, DRTs improve dyslexia detection algorithms’ accuracy and efficiency. These approaches allow for building robust and accurate classification algorithms by detecting discriminative features from neuroimaging, behavioral evaluations, and linguistic analyses. Advanced computational tools may improve dyslexia diagnoses and early intervention efforts, which have significant therapeutic consequences. Multimodality-based DDs enable researchers to understand its causes and develop more reliable diagnoses and therapeutic methods.

The neurological correlates of dyslexia may assist in extracting biomarkers for early identification. The existing therapies can be customized in order to modulate particular brain regions or networks involved in reading difficulties. Behavioral evaluations highlight dyslexic reading deficiencies and assist with intervention development. In addition, they quantify the efficiency of the existing intervention methods. Linguistic investigations may uncover dyslexia-related phonological processing and lexical retrieval impairments. Linguistic deficiencies can influence dyslexia-specific language-based therapies and assistive technologies. Genetic methods reveal dyslexia’s genetics and environmental effects. Genetic risk factors can assist in identifying high-risk dyslexics and providing individualized treatment.

The majority of studies were conducted in controlled settings with limited sample numbers and homogenous participants. Consequently, the results may not apply to varied clinical groups or educational settings. Heterogeneity in research designs, participant characteristics, and outcomes introduces challenges in synthesizing and drawing conclusions. The lack of longitudinal research on dyslexia and feature extraction and selection methodologies hinders understanding its progression and predictive value. Using sensitive data, including neuroimaging scans or genetic information, may raise ethical considerations. Dyslexia detection requires balancing model interpretability and classification performance. Despite improved accuracy, complex machine learning algorithms lack interpretability, limiting clinical value. High-performance interpretable models are crucial for practical implementation. Limited effective fusion techniques and data heterogeneity across modalities reduce the performance of the existing models. Recently developed DL algorithms like CNNs and recurrent neural networks may derive hierarchical representations from complex data sources. These algorithms may find novel biomarkers and enhance DD. Strengthening fusion algorithms across different data modalities may improve dyslexia identification.

To evaluate DRTs, robust assessment and benchmarking frameworks are essential. Standardized assessment measures, publicly accessible datasets, and iterative experimental techniques enable fair comparisons and reveal critical strengths and weaknesses of alternative methodologies. Community-driven competitions and workshops may foster cooperation and innovation in DRT approaches. The benefits and drawbacks of DRTs should be carefully considered to improve the DD model’s efficiency. There should be continuing methodological advancement and validation within the context of dyslexia identification. Researchers may improve the DD model’s accuracy, interpretability, and generalization by strategically using DRT and addressing its limitations. Multimodal fusion, longitudinal assessments, and open science initiatives may overcome shortcomings in the existing methodologies, improve dyslexia diagnosis accuracy, and foster field information sharing and cooperation.

### Limitations and Future Directions

The likelihood of publication bias, the exclusion of non-English language research, and the use of the specified search keywords and databases may have influenced the findings of this review. The inclusion and exclusion criteria may have favored specific research, leading to selection bias. Prioritizing methodologically rigorous or statistically significant studies may have overlooked valuable research with differing methods or outcomes. The quality of neuroimaging or behavioral data may have influence DRTs’ effectiveness in the included studies. Study heterogeneity and methodology variations may have restricted generalizability. The included studies may have demanded substantial computational resources that may affect their implementation in clinical settings.

A lack of high-quality labeled data restricts dyslexia detection research. To overcome this limitation, researchers can employ synthetic data generation or transfer learning to build effective DRTs that perform well with small or unbalanced datasets. Data augmentation and generative models may enrich dyslexia-related neuroimaging datasets. In order to adapt to dyslexia’s distinct neurocognitive processes, future research should integrate DRTs with customized data to develop personalized models. Using multi-modal, longitudinal data of the same and different individuals may assist in developing models, enhancing dyslexia diagnoses and treatments. In high-dimensional, sparse datasets like neuroimaging, sparse PCAs may overcome the limitations of traditional PCAs. Sparse PCAs prioritize essential features to reduce noise while maintaining interpretability. Sparse PCAs and CNNs, which excel at learning spatial hierarchies, may enhance the performance of dyslexia detection models. This hybrid technique may identify dyslexia-related brain problems by capturing reduced-dimensionality patterns and local image features. In the future, lightweight DRTs can be developed to improve DD effectiveness and support educational and healthcare settings with limited computational resources.

## 5. Conclusions

In this review, the authors highlighted the crucial role of DRTs in detecting dyslexia using multiple modalities. The comprehensive analysis of 39 articles yielded the significance of DRTs in improving ML- and DL-based dyslexia identification. The findings underscore the critical contribution of the included studies in extracting and integrating unique features in DD. The authors identified essential dyslexia features extracted from different modalities, including MRI, EEG, eye movements, and handwritten images. They presented the notable challenges and limitations of the included studies. The lack of generalizability, black-box nature of ML and DL models, high computation costs, limited datasets, and data privacy hinder the development of advanced DD models. These limitations should be addressed in order to build optimal DD models. Timely detection of dyslexia demands a low-cost and highly interpretable model. Healthcare and educational centers can benefit from these models to support DIs and improve their quality of life. This review encompasses studies with unique methodologies and data samples. The heterogeneity of the included studies may influence the reliability of the review outcomes. The inclusion and exclusion criterion may overlook significant dyslexia identification studies. There may be publication biases, potentially skewing the overall findings. Addressing these limitations can provide substantial insights into DRTs and their role in identifying dyslexia in its earlier stages.

## Figures and Tables

**Table 1:** Inclusion and exclusion criteria.

Inclusion	Exclusion
Articles published in conferences and peer-reviewed journals indexed in Scopus, PubMed, and Web of Science.	Book chapters, editorial letters, and dissertations.
No date restrictions.	Non-English language studies.
Original research studies related to DD.	Animal studies, case reports, and non-research articles.
Studies based on DRTs, including MRI, EEG, handwritten images, and behavioral assessments.	Study outcomes irrelevant to DD or not covering DRTs.
Studies employing standard performance metrics, including accuracy, sensitivity, specificity, and F1-score.	—

Search String = (“Dyslexia” OR “Reading disability” OR “Developmental dyslexia” OR “Learning disorder”) AND (“Detection” OR “Diagnosis” OR “Identification” OR “screening”) AND (“Feature extraction” OR “Feature selection” OR “Dimensionality reduction” OR “Machine learning” OR “Deep learning” OR “Artificial intelligence” OR “PCA” OR “t-SNE” OR “SVD” OR “ICA”, “UMAP”, OR “LDA”).

**Table 2:** Quality assessment techniques.

Criterion	Score 0 (Poor)	Score 1 (Fair)	Score 2 (Good)	Score 3 (Excellent)
Study design	Descriptive	Observational	Controlled	Randomized
Sample size	<30	30–50	51–100	>100
Data analysis	Inappropriate	Basis analysis	Appropriate	Advanced
Bias mitigation	No strategies	Minimal strategies	Some strategies	Comprehensive strategies
Evaluation metrics	No evaluation metrics	Minimal set of metrics	Minimal set of metrics and comprehensive analysis	Comprehensive evaluation metrics and analysis

**Table 3:** PCA-based DD models.

Authors	Datatype	Classifier	Dataset Size (Number of Individuals)	Performance	Limitations
Al-Barhamtoshy and Motaweh (2017) [17]	EEG	SVM	80	Accuracy: 81.06%, precision: 62%, recall: 100%, F1-score: 76.64%	Computational resources, user training, and system usability are crucial for effective implementation and acceptance.
Asvestopoulou et al. (2019) [18]	Eye movements	SVM	135	Accuracy: 97%	The model’s performance is based on the quality of eye-tracking data.
Appadurai and Bhargavi (2019) [19]	Eye movements	SVM with PSO	185	Accuracy: 96%	High computational costs and performance may vary in novel datasets.
Raatikainen et al. (2021) [20]	Eye movements	SVM	165	Accuracy: 89.7%, recall: 84.8%	The transition matrix reduced the classification accuracy of the model.
Christodoulides et al. (2022) [21]	EEG	RF	26	Accuracy: 97%, sensitivity: 96%	The variations in EEG signal may limit the model’s performance.
Parmar and Paunwala (2023) [22]	EEG	SVM	53	Accuracy: 79.3%	Obtained a low accuracy of 77.3% due to the limited functionality of PCA.
Liyakathunisa et al. (2023) [23]	Behavioral data	NN	77	Accuracy: 95.3%	The model performance was based on a web-based game.
Parmar and Paunwala (2023) [24]	EEG	SVM	391	Average accuracy: 98.72%	The shortcomings of the SVM model may affect the classification accuracy.
Zaree et al. (2023) [25]	ERP	Ensemble learning	121	Accuracy: 87.5%, sensitivity: 81.2%	A low performing classifier may influence the overall classification performance.
Zhong et al. (2023) [26]	Handwritten images	XGBoost	207	Accuracy: 81.06%, sensitivity: 74.27%, specificity: 82.71%, AUC: 0.79	Variations in the handwritten images may affect the model’s generalizability.
El-Hmimdi et al. (2024) [27]	Eye movements	CNN	222	Precision: 80.2%, recall: 75.1%	Lack of interpretability may cause challenges for clinicians.
Shalileh et al. (2024) [28]	Eye movements	Multi-layer perceptron	144	Precision: 0.93, recall: 0.93, F1-score: 0.93, AUC: 0.98	The limited functionality of a multi-layer perceptron may affect the model performance in real-time settings.

**Table 4:** CNN-based DD models.

Authors	Datatype	Classifier	Dataset Size (Number of Individuals)	Performance	Limitations
Usman and Muniyandi (2020) [29]	MRI	CNN	45	Accuracy: 73.2%	The model’s performance may differ in less resource-intensive settings.
Tomaz Da Silva et al. (2021) [30]	MRI	CNN	32	Accuracy: 94.3%	Lack of data augmentation technique.
Sangeetha et al. (2022) [31]	MRI	NN	58	Accuracy: 99.8%, recall: 91.6%, precision: 92.3%	The computation cost may affect the model’s implementation.
Harismithaa and Sudha (2022) [32]	MRI	Convolution-LSTM	31	Accuracy: 98.3%	Lack of generalizability in novel datasets.
Sasidhar et al. (2022) [33]	Handwritten images	Residual NN	Normal: 78,275 Reversal: 52,196 Corrected: 8.029	Accuracy: 97.6%	Residual NN model limitations, including complexity and overfitting, may reduce the model’s performance.
Ileri et al. (2022) [34]	EOG signals	CNN	43	Horizontal EOG accuracy: 98.7%, vertical EOG accuracy: 80.94%	Limitations of the one-dimensional CNN model may reduce the model’s performance on the novel dataset.
Kothapalli et al. (2022) [35]	MRI and EEG	Decision tree	75	Accuracy: 92.2%, recall: 91.9%, F1-Score: 96.6%, AUC: 0.98.	The performance of base models may affect the model’s performance in novel datasets.
Jasira and Laila (2023) [36]	Handwritten images	LSTM	Normal: 78,275 Reversal: 52,196 Corrected: 8.029	Accuracy: 89.1%	The model’s performance is limited to the English language.
Liu et al. (2024) [37]	Handwritten images	LSTM	1064	Accuracy: 85%, sensitivity: 83.3%, specificity: 86.4%, AUC: 0.90	Black-box nature of the DL model may reduce the interpretation of the outcomes.

## Data Availability

Data sharing is not applicable for this study.
